# Near theoretical ultra-high magnetic performance of rare-earth nanomagnets *via* the synergetic combination of calcium-reduction and chemoselective dissolution

**DOI:** 10.1038/s41598-018-33973-z

**Published:** 2018-10-23

**Authors:** Jimin Lee, Tae-Yeon Hwang, Hong-Baek Cho, Jongryoul Kim, Yong-Ho Choa

**Affiliations:** 1 Department of Materials Science and Chemical Engineering, Hanyang University, 55, Hanyangdaehak-ro, Sangnok-gu, Ansan-si, Gyeonggi-do 15588 Korea; 2 Department of Fusion Chemical Engineering, Hanyang University, 55, Hanyangdaehak-ro, Sangnok-gu, Ansan-si, Gyeonggi-do 15588 Korea

## Abstract

Rare earth permanent magnets with superior magnetic performance have been generally synthesized through many chemical methods incorporating calcium thermal reduction. However, a large challenge still exists with regard to the removal of remaining reductants, byproducts, and trace impurities generated during the purifying process, which serve as inhibiting intermediates, inducing productivity and purity losses, and a reduction in magnetic properties. Nevertheless, the importance of a post-calciothermic reduction process has never been seriously investigated. Here, we introduce a novel approach for the synthesis of a highly pure samarium-cobalt (Sm-Co) rare earth nanomagnet with near theoretical ultra-high magnetic performance *via* consecutive calcium-assisted reduction and chemoselective dissolution. The chemoselective dissolution effect of various solution mixtures was evaluated by the purity, surface microstructure, and magnetic characteristics of the Sm-Co. As a result, NH_4_Cl/methanol solution mixture was only capable of selectively rinsing out impurities without damaging Sm-Co. Furthermore, treatment with NH_4_Cl led to substantially improved magnetic properties over 95.5% of the *M*_*s*_ for bulk Sm-Co. The mechanisms with regard to the enhanced phase-purity and magnetic performance were fully elucidated based on analytical results and statistical thermodynamics parameters. We further demonstrated the potential application of chemoselective dissolution to other intermetallic magnets.

## Introduction

In the past few decades, exchange-coupled nanomagnets comprising both magnetically hard- and soft-phases have been intensively studied within the permanent magnet industry, due to their exceptional magnetic characteristics beyond conventional magnetic compounds^1^. Through a theoretical study, Skomski and Coey demonstrated an expected value as high as 120 MG·Oe for the maximum energy product of a hard/soft-coupled composite magnet, while Nd_2_Fe_14_B as a superior hard magnet yielded ~56 MG·Oe^2,3^. This excellent magnetic performance could be attributed to a unique interaction between two different phases, referred to as the “*exchange-coupling effect*”^4^. To obtain hard/soft exchange-coupled nanocomposites, many fabrication methods have been reported: one of the simplest chemical routes involves surface treating a hard nanomagnet such as plating of the soft phase^5,6^, sputtering^7^, and the glyoxylate precursor method^8^.

As hard nanomagnets, rare earth element based metallic phases, including neodymium-iron-boron (Nd-Fe-B), samarium-cobalt (Sm-Co), and samarium-iron-nitride (Sm-Fe-N), are expected to experimentally exhibit the strongest exchange-coupling behavior due to their exceptionally high coercivity and energy product^9^. Among magnetic hard materials, Sm-Co (*e.g*., Sm_2_Co_17_, SmCo_5_) nanostructures have been prepared through a variety of chemical methods such as sol-gel^10^, co-precipitation^11^, electrospinning^12^, and electrodeposition^13^, followed by a subsequent reduction-diffusion (R-D) process. Recently, studies with regard to control over the microstructures of single-phased nanomagnets with either a near single-domain size or a high-anisotropic feature are being developed in order to achieve further enhanced magnetic properties^14,15^. One way to precisely control the fibre diameter of magnetic structures is to employ electrospinning process. When the fibre dimension reaches the single-domain size of a given magnet (*e.g*., Sm_2_Co_17_: sub-micron-scale), the theoretical maximum coercivity can be achieved^16^.

The reduction process is an indispensable step in all the chemical approaches to prepare rare earth magnets from oxides. Because rare earth elements possess a highly negative reduction potential (*e.g*., Sm^3+^/Sm = −2.41 eV; while transition metal, Co^2+^/Co = −0.28 eV) and a low free energy for oxidation (*e.g*., Sm = −5.73 × 10^5^ J at 25 °C), rare earth oxides are extremely stable and are difficult to reduce to their metallic phase under H_2_ conditions^12,17,18^. Calcium (Ca) granule or calcium hydride (CaH_2_) powder with an oxidation energy (*e.g*., Ca = −6.04 × 10^5^ J at 25 °C) lower than all other rare earth metals has enabled the reduction, leading to a production of metallic rare earth magnets and oxidized Ca (CaO)^19^. To eliminate the unconsumed or residual calcium phases, a post-calciothermic reduction process was imperative; thus far, a dilute acidic solution and/or deionized water have been conventionally used for rinsing out leftover reductants^20–25^. However, a byproduct unavoidably formed, resulting in poor magnetic properties of the resultant nanomagnet. As the residue and water reacted intensely with the liberation of an enormous amount of heat, Ca/CaO formed a water-insoluble calcium compound and it remained as a non-magnetic product. Moreover, H_2_ gas was produced vigorously and induced proton (H^+^) formation in acidic solution, causing serious damage to the nanomagnets. In the worst case, magnetic phase decomposition could occur^26^.

Thus, after the well-controlled synthesis of the nanomagnets, the existence and unsuccessful removal of unwanted impurities led to inferior magnetic properties as well as surface damage to the hard magnetic nanomaterials. The surface defects further resulted in difficult exchange-coupling interactions on hard- and soft-magnetic inter-phases. However, most previously reported studies focused only on the synthetic results without covering the loss in magnetic properties induced by side products and the interaction between byproducts and treated solutions^22,27–29^. Interestingly, Wang *et al*. proposed a novel washing route (*i.e*., ethyl alcohol-water; two-step process) for the synthesis of Nd-Fe-B nanoparticles with excellent magnetic properties; however, they could not deviate from the problems of oxidation and the formation of serious defects on the surface of the metallic magnetic phase^30^. To the best of our knowledge, there have not been detailed studies addressing the effects of a chemoselective dissolving solution on the surface characteristics and magnetic properties of nanoscale magnetic structures prepared by the R-D process.

**Figure 1:**
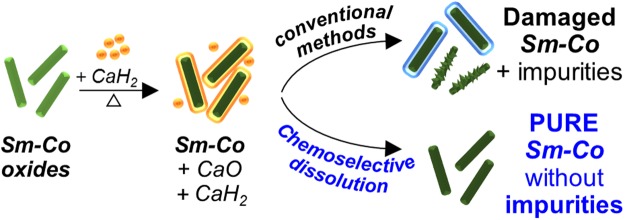
Illustration of the experimental procedure to obtain Sm_2_Co_17_ nanofibres.

Here, 1-D highly pure samarium-cobalt nanostructures with near theoretical ultra-high magnetic performance were synthesized *via* consecutive electrospinning, calcium thermal reduction, and chemoselective dissolution. The chemoselective effects of various conventional solutions were evaluated and fully discussed according to as-treated rare earth magnetic Sm_2_Co_17_ nanofibres and their magnetic properties and surface microstructural characteristics. Moreover, the applicability of the most efficient selective-dissolving solution to other rare earth based magnetic phases (*e.g*., SmCo_5_ and Nd_2_Fe_14_B system) was also discussed in order to obtain high purity, outstanding magnetic performance, and to demonstrate further potential as a raw material applied to an exchange-coupled magnet. A graphical summary of our experimental procedure can be seen in Fig. 1.

## Results and Discussion

### Preparation of solutions for chemoselective dissolution

Solutions for chemoselective dissolution must fulfill the following condition: possess a high Ca/CaO solubility or fully react with these calcium compounds. Pure alcohols (*e.g*., ethanol, methanol) were not selected because these solutions cannot react with the byproducts. Additionally, CaO and CaH_2_ possess extremely low solubility in alcohols^31,32^. Ethylene glycol and glycerol are potentially usable; however, these chemicals were excluded due to their sluggish reactivity with CaO^33^. Distilled water and dilute acidic solutions (*e.g*., acetic acid, hydrochloric acid (HCl)) have already been widely reported as traditional washing solutions^20,21,34^. In this study, the use of a strong acid was not considered even if it was largely diluted, as it could lead to the serious corrosion of metal magnetic compounds. CaO was reported to be soluble in water-based sugar solutions. For example, a 34 *w/v*% sucrose solution dissolved 9.45 mass % of CaO at 25 °C^35^. Glucose, rhamnose, lactose, and raffinose could be employed as sugars; however, these powders exhibited relatively low solubility compared to sucrose, which possessed a solubility of 201 g/100 mL of water^36^. The application of a NH_4_Cl-methanol mixed solution after calcium-assisted thermal reduction has been reported for the synthesis of a single element magnet (*i.e*., α-Fe) and some nonmagnetic materials (*i.e*., LaNiO_2_, La_2_CuO_4_)^34,37–42^. However, only a few papers have been published for binary or ternary alloy magnets^27,43,44^. All things considered, four different solutions were selected: pure distilled water, 0.1 M dilute acetic acid solution, 85 *w/v*% sucrose solution, and 0.1 M of NH_4_Cl in methanol.

### Phase, surface characteristics, and magnetic properties of treated Sm-Co nanofibres using different solution treatment

**Figure 2:**
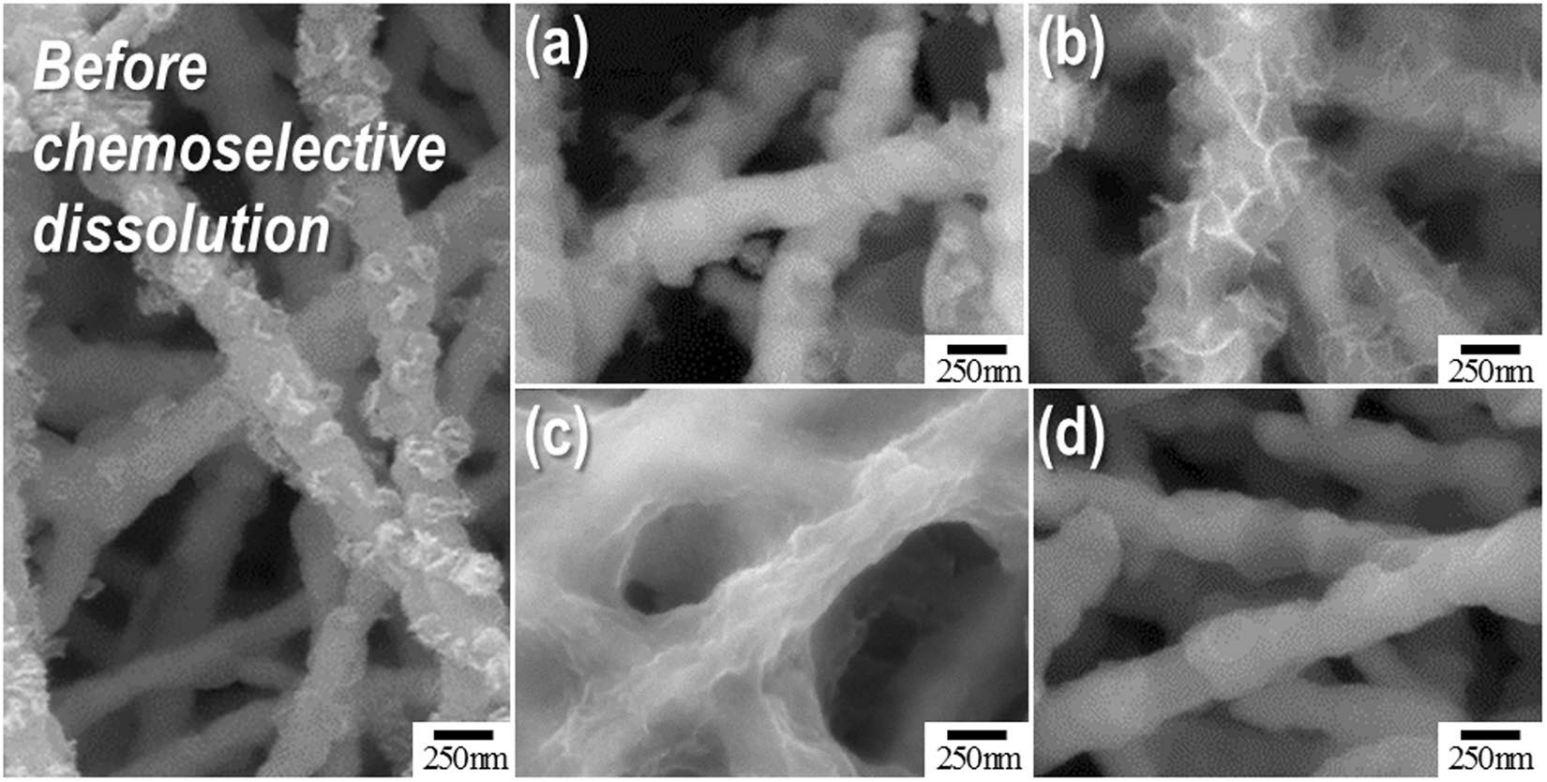
FE-SEM micrographs of the as-reduced Sm_2_Co_17_ nanofibres obtained prior to and after washing with various solutions: (**a**) pure distilled water, (**b**) 0.1 M dilute acetic acid in water, (**c**) 85 *w*/*v*% of sucrose in water, and (**d**) 0.1 M NH_4_Cl in methanol.

Figure 2 shows the surface morphology of Sm-Co nanofibres obtained after washing and drying under various dissolution conditions. As-reduced samples were coated with a rough layer of CaO and residual CaH_2_ particles. There was not a distinct morphology difference between samples obtained prior to and after dissolution using only distilled water (Fig. 2(a)). Different layer-morphologies were observed when samples were treated with dilute acid or sucrose solutions, as can be seen in Fig. 2(b,c). Interestingly, nanofibres with a smooth surface morphology were obtained when a NH_4_Cl/methanol solution was employed (Fig. 2(d)).

**Figure 3:**
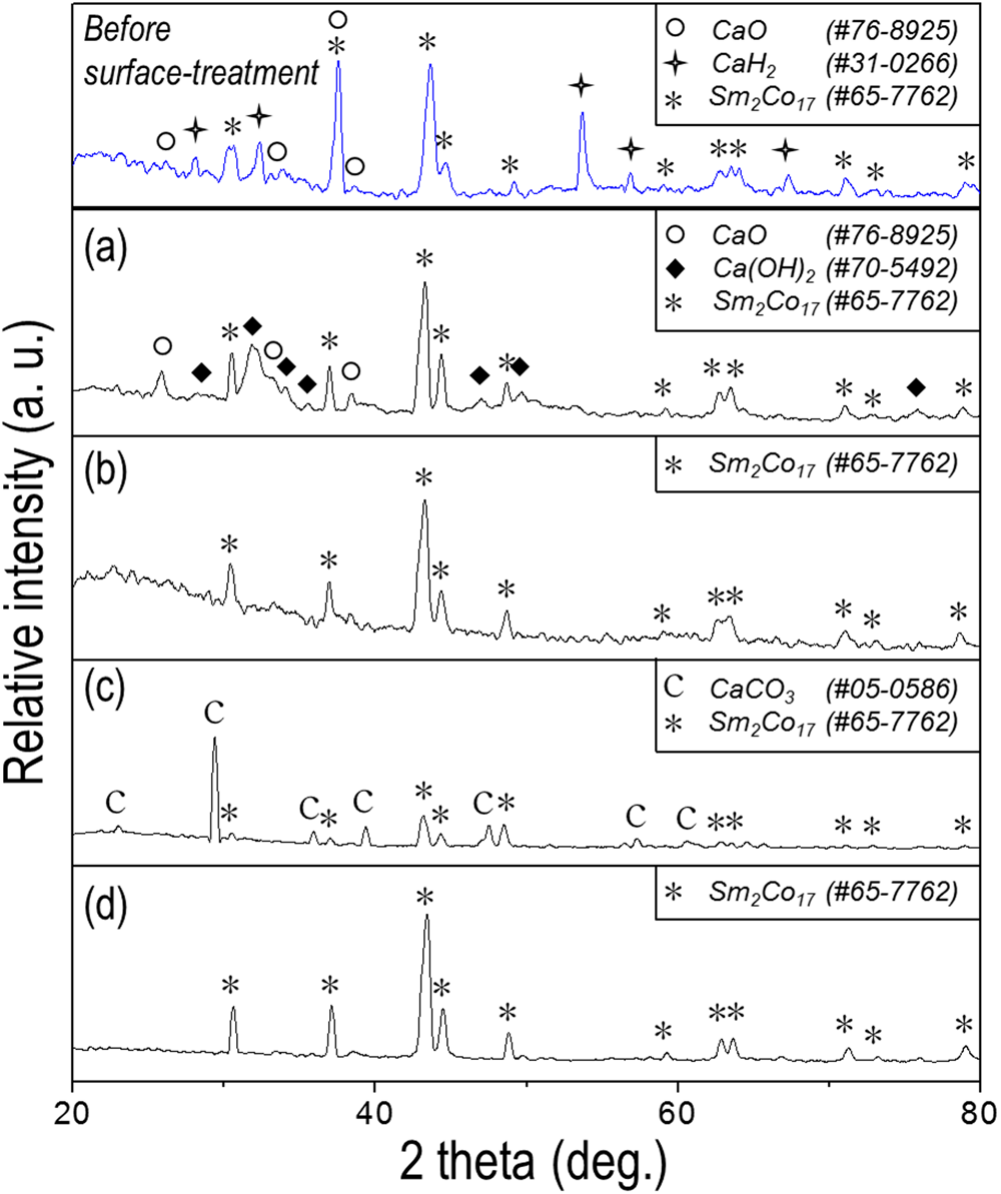
XRD patterns of the as-reduced Sm_2_Co_17_ nanofibres obtained prior to and after washing with: (**a**) pure distilled water, (**b**) 0.1 M dilute acetic acid in water, (**c**) 85 *w*/*v*% of sucrose in water, and (**d**) 0.1 M NH_4_Cl in methanol.

Powder X-ray diffraction patterns of the treated Sm-Co nanofibres can be seen in Fig. 3. Prior to treatment, the unreacted CaH_2_, CaO, and pure Sm_2_Co_17_ phases were observed. As CaO and residual CaH_2_ reacted with distilled water to generate calcium hydroxide (Ca(OH)_2_), the diffraction patterns of this insoluble phase and some CaO were obtained (Fig. 3(a), see the result with different the number of washing in Fig. S6)^45^. When aqueous acid and NH_4_Cl/methanol solutions were applied, a clear Sm_2_Co_17_ diffraction pattern was observed in Fig. 3(b,d). However, a broadened pattern throughout low angles between the range of 20-40° was also visible in Fig. 3(b), presumed that there was damage on Sm-Co, such as phase amorphization. Meanwhile, a high intensity water-insoluble CaCO_3_ phase was observed with Sm_2_Co_17_ exhibiting low intensity in Fig. 3(c). This could be attributed to side reactions between calcium compound and saccharose solutions^35,46–48^.

**Figure 4:**
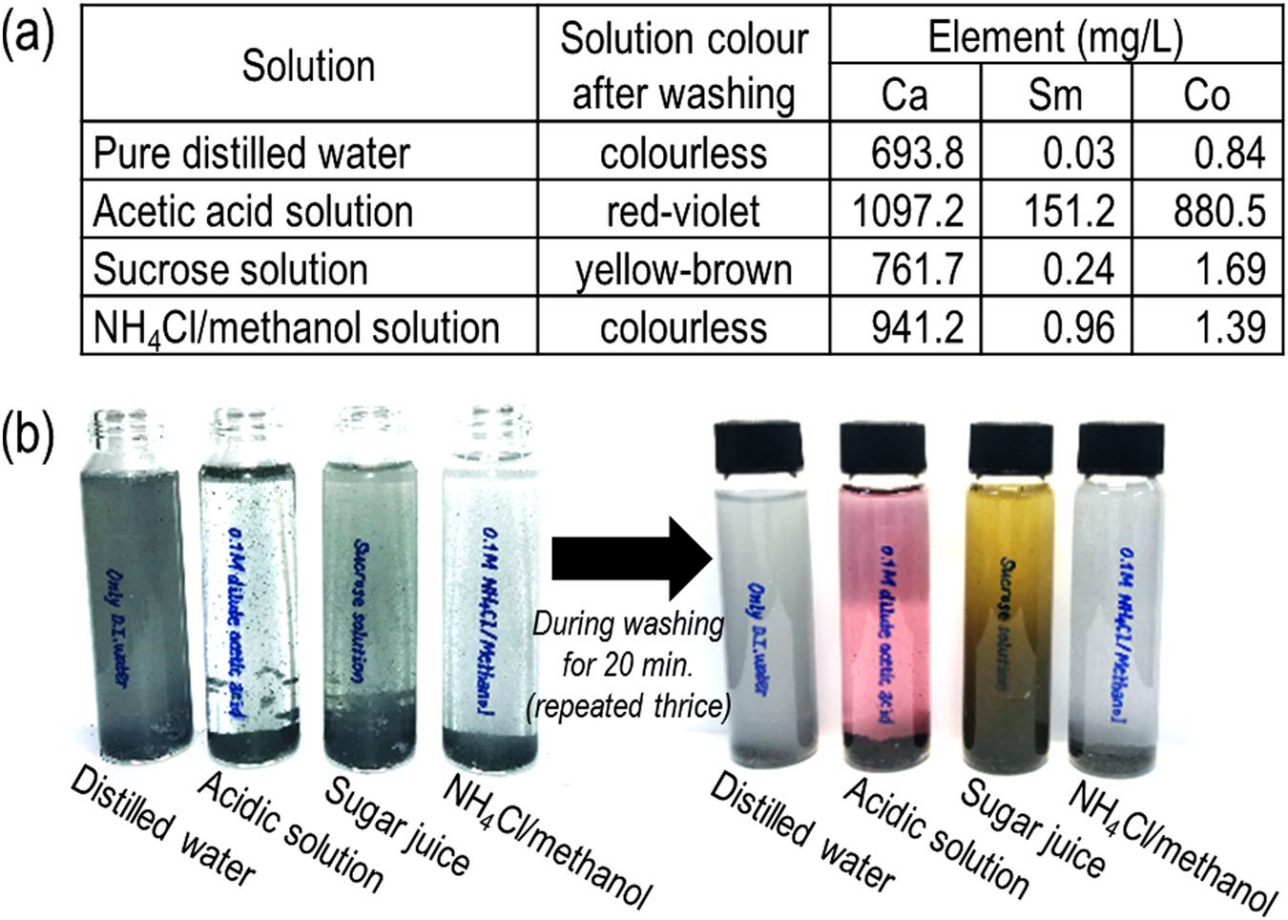
(**a**) ICP-OES analysis data of the elemental concentration in each solution (error range of ±~0.1%) and (**b**) photographs of the obtained solutions during the washing process.

The best dissolution precursor candidate should be able to selectively rinse away Ca without dissolving Sm or Co. To confirm this process, the concentrations of 3 elements, Ca, Sm, and Co in each solution obtained during the dissolution process were determined by ICP-OES as having an error of ±~0.1% (Fig. 4(a)). For all the given solution conditions, over 600 mg/L of Ca was successfully removed. However, a considerable amount of Sm and Co, both over 150 mg/L, was also detected in the dilute acetic acid solution. It is considered that the acidic solution had an undesirable impact on the physical characteristics (*i.e*., structural and magnetic properties) of Sm-Co. There was also a colour change in some solutions (Fig. 4(b)): the dilute acetic acid solution and sucrose juice turned red and yellow, respectively, while distilled water and NH_4_Cl/methanol solution remained colourless. This may have been related to the existence of hydrogen ions, H^+^, during the rinsing process. It was reported that an excess amount of H^+^ in an acidic solution changed the colour of the cobalt(II) acetate ionic complex to pinkish red, which affected the colour change of metallic Sm-Co. Colourless samarium acetate, Sm(CH_3_COO)_3_·*x*H_2_O also formed^49^. The reaction of calcium with the saccharose solution yielded viscous yellow-brown solutions while monocalcium saccharates were dispersed^48^.

**Figure 5:**
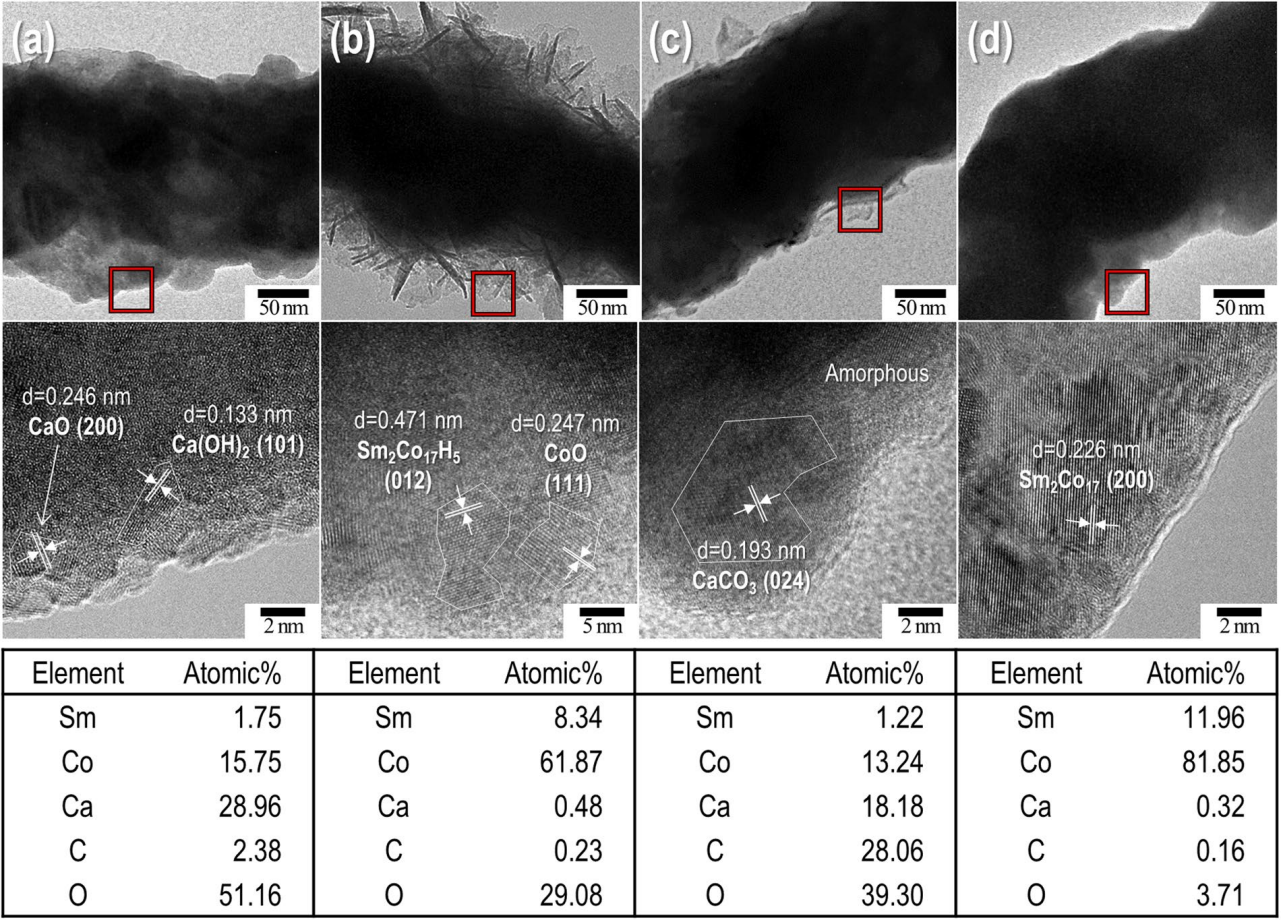
TEM images and HR-TEM micrographs (as indicated by the red square) of Sm_2_Co_17_ nanofibres prepared under different washing conditions: (**a**) pure distilled water, (**b**) 0.1 M dilute acetic acid in water, (**c**) 85 *w*/*v*% of sucrose in water, and (**d**) 0.1 M NH_4_Cl in methanol. The chemical compositions of the constituents were determined *via* TEM-EDS with an error of ±~1%.

To investigate the microstructure and phase of the surface, the as-rinsed Sm_2_Co_17_ fibres were analysed *via* TEM (see Fig. 5). With regard to the water-treated sample, altocumulus-like layers consisting of Ca(OH)_2_ (JCPDS No.70-5492) and CaO (JCPDS No.76-8925) were observed (Fig. 5(a)). The filiform layer produced from the acidic solution corresponded to Sm_2_Co_17_H_5_ (JCPDS No.79-9700) (Fig. 5(b)). In Fig. 5(c), there were several amorphous layers and CaCO_3_ (JCPDS No.05-0586) on the surface of the sucrose-treated Sm_2_Co_17_ nanofibres, which may be attributed to the unexpected reaction between CaO/Ca(OH)_2_ and the sugar-derivatives. It was believed that a little calcium saccharate, Ca(C_12_H_22_O_11_)_2_, with a very long carbon chain, remained on the surface as an amorphous layer^50^. Naked Sm_2_Co_17_ crystals (JCPDS No.65-7762) were clearly seen on the surface of the treated fibres when the NH_4_Cl/methanol solution was employed, implying no damage occurred to the resulting fibres (Fig. 5(d)). The elemental profile of the surface for Sm, Co, Ca, carbon (C), and oxygen (O) was investigated using TEM-EDS and the results were in accordance with the formed phases in each solution. There was an appreciable quantity of Ca in the water-treated Sm_2_Co_17_ samples, indicating that distilled water was inadequate to remove CaH_2_ and CaO. It turned out that the sucrose solution was not suitable as a treatment solution due to a large portion of C induced from the CaCO_3_ phase as in the water-treatment case. In the acid-treated Sm_2_Co_17_, there was a small amount of Ca; however, a considerable amount of O was observed. On the contrary, the TEM-EDS data confirmed the presence of Sm and Co without any impurities including Ca and C between the standard error range of ±~1% for NH_4_Cl/methanol-treated Sm_2_Co_17_ sample.

**Figure 6:**
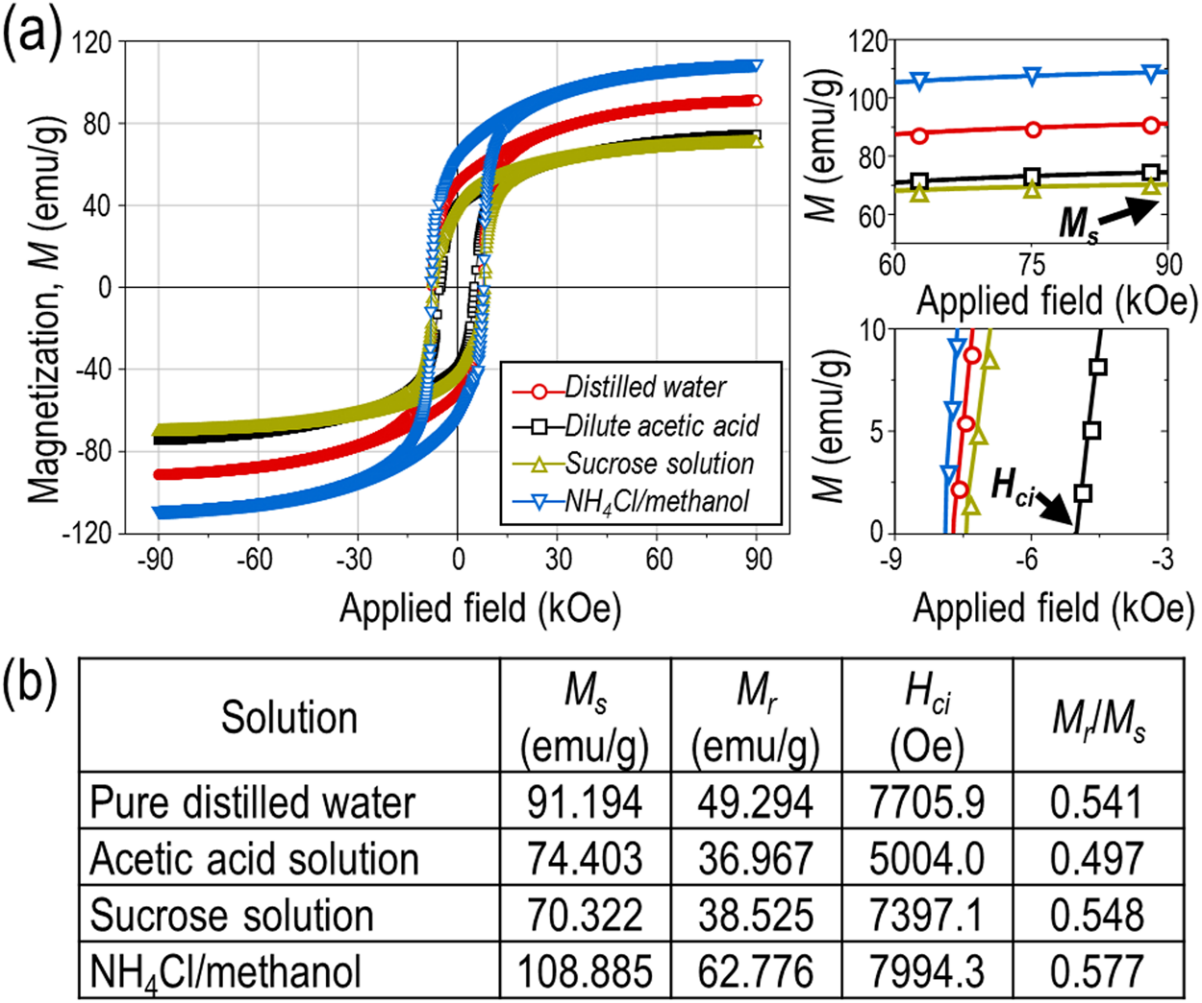
(**a**) Magnetic hysteresis loops of the pretreated Sm_2_Co_17_ nanofibres with different solution treatments. (**b**) The corresponding saturation magnetisation (*M*_*s*_), remanence (*M*_*r*_), coercivity (*H*_*ci*_), and squareness (*M*_*r*_/*M*_*s*_) values for the as-washed Sm_2_Co_17_.

The magnetic properties of the Sm_2_Co_17_ powder samples were investigated using PPMS without compaction, magnetic alignment, and sintering. The magnetic hysteresis loops as a function of the treatment solution can be seen in Fig. 6(a). The corresponding saturation magnetisation (*M*_*s*_), remanence (*M*_*r*_), intrinsic coercivity (*H*_*ci*_), and squareness (*M*_*r*_/*M*_*s*_) values are given in Fig. 6(b). Despite utilization of the same Sm_2_Co_17_ as a starting powder material, the magnetic properties varied depending on the solution used during the dissolution treatment because the *H*_*ci*_ and *M*_*s*_ values were strongly affected by the purity of the phase^51^. It was known that pure bulk Sm_2_Co_17_ possessed a high *M*_*s*_ (*M*_*bulk*_) of 114.0 emu/g (calculated from 1.0 MA/m with a density of Sm_2_Co_17_ = 8.769)^52^. For the NH_4_Cl/methanol-treated nanofibres, *M*_*s*_ was found to be 108.9 emu/g, which was within 95% of the theoretical value for Sm_2_Co_17_. It was worth mentioning that this difference (~5 emu/g) was negligible due to a magnetically “dead” layer on the surface of the Sm-Co fibres within the nanoscale regime^53,54^. On the contrary, *M*_*s*_ deteriorated as low as 62% of the original value in the other cases. For the as-washed samples treated with distilled water or sucrose juice, the decrease in *M*_*s*_ resulted from a considerable amount of impurities including CaO and Ca(OH)_2_, while *H*_*ci*_ was not affected by these diamagnetic materials^55^. With regard to the surface treatment utilizing an acidic solution, a remarkable *H*_*ci*_ drop occurred due to the formation of a thin Sm_2_Co_17_H_*x*_ layer with soft magnetic characteristics. Besides, interstitial hydrogen in the magnetic phase reduced the anisotropy field, leading to a further drastic decrease in *H*_*ci*_^22,56,57^. Therefore, it could be concluded that the NH_4_Cl/methanol solution was the most suitable chemoselective dissolution solution leading to high magnetic performance 1-D Sm_2_Co_17_ nanostructures.

### Effect of chemoselective dissolution induced-by different reaction mechanisms

All of the aforementioned dissolution reactions were evaluated in terms of a spontaneous chemical reaction with a lower Gibbs free energy.

#### Distilled water

The CaH_2_ phase fully reacted with H_2_O due to its high reactivity, leading to the formation of insoluble Ca(OH)_2_. As a result, it could be detected as a layer in the SEM and TEM micrographs:

1 $$\begin{array}{rcl}{{\rm{CaH}}}_{2}+2{{\rm{H}}}_{2}{\rm{O}} & \to  & {\rm{Ca}}{({\rm{OH}})}_{2}(\,\downarrow \,)+2{{\rm{H}}}_{2}({\rm{g}})\ldots \ldots ({{\rm{\Delta }}{\rm{G}}}_{298{\rm{K}}}^{0}=-\,68.354\,\mathrm{kcal}),\\  &  & ({\rm{\Delta }}{H}_{298K}^{0}=-\,56.706\,\mathrm{kcal})\end{array}$$

2 $$\begin{array}{rcl}{\rm{CaO}}+{{\rm{H}}}_{2}{\rm{O}} & \to  & {\rm{Ca}}{({\rm{OH}})}_{2}(\,\downarrow \,)\ldots \ldots ({{\rm{\Delta }}{\rm{G}}}_{298{\rm{K}}}^{0}=-\,13.815\,\mathrm{kcal}),\\  &  & ({\rm{\Delta }}{H}_{298K}^{0}=-\,15.571\,\mathrm{kcal})\end{array}$$

where the standard Gibbs free energy (∆G^0^) and the enthalpy change (∆H^0^) were calculated using the *HSC Chemistry* software while assuming the entire reaction temperature was 25 °C^58^. The produced Ca(OH)_2_ layer located on the outer Sm_2_Co_17_ surface could readily coat the surface preventing further reaction of residual CaO with water in the deeper internal body. Thus, CaO and Ca(OH)_2_ phases were observed in the X-ray diffraction pattern and micrographs. A negative ∆H for Equations (1 and 2) indicated that the two reactions were exothermic and produced a lot of heat.

#### Dilute acetic acid solution

When dilute acetic acid was utilized, CaO-CaH_2_ and the dilute acetic acid could react as follows:

3 $$\begin{array}{c}{{\rm{CaH}}}_{2}+2[{({{\rm{CH}}}_{3}{\rm{COO}})}^{-}{{\rm{H}}}^{+}]\to {\rm{Ca}}{({{\rm{CH}}}_{3}{\rm{COO}})}_{2}({\rm{aq}})+2{{\rm{H}}}_{2}({\rm{g}})\\ \,\,\ldots \ldots \ldots \ldots ({{\rm{\Delta }}{\rm{G}}}_{298{\rm{K}}}^{0}=-\,86.372\,\mathrm{kcal}),({\rm{\Delta }}{H}_{298K}^{0}=-\,87.500\,\mathrm{kcal})\end{array}$$

4 $$\begin{array}{c}{\rm{CaO}}+2[{({{\rm{CH}}}_{3}{\rm{COO}})}^{-}{{\rm{H}}}^{+}]\to {\rm{Ca}}{({{\rm{CH}}}_{3}{\rm{COO}})}_{2}({\rm{aq}})+{{\rm{H}}}_{2}{\rm{O}}({\rm{l}})\\ \,\,\ldots \ldots \ldots \ldots ({{\rm{\Delta }}{\rm{G}}}_{298{\rm{K}}}^{0}=-\,31.833\,\mathrm{kcal}),({\rm{\Delta }}{H}_{298K}^{0}=-\,46.365\,\mathrm{kcal})\end{array}$$

Compared to the case in distilled water, the large exothermic enthalpy of Equations (3 and 4) accelerated the generation of activated H_2_, resulting in the hydrogenation of Sm-Co nanostructures^57,59^. Although the hydride phase was indistinguishable in the X-ray diffraction pattern (see Fig. S2), the deteriorated magnetic properties provided conclusive evidence for the formation of Sm_2_Co_17_H_*x*_ with a small *H*_*ci*_. Also, due to the lower ionization potential of samarium and cobalt in water compared to hydrogen, remaining proton ions (H^+^) induced from the acid could also affect the Sm-Co alloy leading to a release of Sm^3+^ and Co^2+ ^^60^. These metal ions could compose the ionic complex, and cobalt acetate could form amorphous cobalt(II) oxide as Equation (5)^61^. A large amount of the oxygen in Fig. 5(b) was believed to be due to an amorphous CoO phase.

5 $${\rm{Co}}{({{\rm{CH}}}_{3}{\rm{COO}})}_{2}\cdot 4{{\rm{H}}}_{2}{\rm{O}}\to {\rm{CoO}}(\downarrow )+2{{\rm{CH}}}_{3}{\rm{COOH}}+3{{\rm{H}}}_{2}{\rm{O}}$$

#### Sugar solution

CaO dissolves in sugar solution forming calcium saccharate which is soluble in water^62^:

6 $$\begin{array}{rcl}n{{\rm{CaH}}}_{2}+{{\rm{C}}}_{12}{{\rm{H}}}_{22}{{\rm{O}}}_{11} & \to  & {{\rm{Ca}}}_{n}{{\rm{C}}}_{12}{{\rm{H}}}_{22}{{\rm{O}}}_{11}({\rm{aq}})+n{{\rm{H}}}_{2}({\rm{g}})\\  &  & \,\ldots \ldots \ldots \ldots ({{\rm{\Delta }}{\rm{G}}}_{298{\rm{K}}}^{0}=-\,5.806\,\mathrm{kcal})\end{array}$$

7 $$\begin{array}{rcl}n{\rm{CaO}}+{{\rm{C}}}_{12}{{\rm{H}}}_{22}{{\rm{O}}}_{11} & \to  & {{\rm{Ca}}}_{n}{{\rm{C}}}_{12}{{\rm{H}}}_{22-2n}{{\rm{O}}}_{11}({\rm{aq}})+n{{\rm{H}}}_{2}{\rm{O}}({\rm{l}})\\  &  & \,\ldots \ldots \ldots \ldots ({{\rm{\Delta }}{\rm{G}}}_{298{\rm{K}}}^{0}=-\,6.492\,\mathrm{kcal})\end{array}$$

It was reported that greater CaO solubility could be obtained with a higher sugar concentration in solution due to the formation of additional calcium saccharate^35^. Considering a sucrose with a melting temperature greater than 160 °C and its solubility (*i.e*., 201 g/100 mL of water), a sucrose concentration of 85 *w*/*v*% in water was selected as an appropriate concentration^36,63^. However, as shown in Fig. 5(c), the organic material caused CaCO_3_ formation: A lot of heat, produced from the exothermic reactions between calcium compounds and water (Equations (1 and 2)), led to CO_2_ gas generation followed by partial thermal-breakdown of the sugar (C_12_H_22_O_11_) to give the organic structure containing one carbon atom fewer than the parent counterpart. When CO_2_ gas was applied to the residual calcium, CaCO_3_ is easily formed (Equations (8 and 9))^47^.

8 $$\begin{array}{rcl}{\rm{CaO}}+{{\rm{CO}}}_{2}({\rm{g}}) & \to  & {{\rm{CaCO}}}_{3}(\,\downarrow \,)\ldots \ldots \ldots \ldots ({{\rm{\Delta }}{\rm{G}}}_{298{\rm{K}}}^{0}=-\,31.172\,\mathrm{kcal}),\,\\  &  & ({\rm{\Delta }}{H}_{298K}^{0}=-\,42.585\,\mathrm{kcal})\end{array}$$

9 $$\begin{array}{rcl}{\rm{Ca}}{({\rm{OH}})}_{2}+{{\rm{CO}}}_{2}({\rm{g}}) & \to  & {{\rm{CaCO}}}_{3}(\,\downarrow \,)+{{\rm{H}}}_{2}{\rm{O}}({\rm{l}})\ldots \ldots \ldots \ldots ({{\rm{\Delta }}{\rm{G}}}_{298{\rm{K}}}^{0}=-\,17.357\,\mathrm{kcal}),\\  &  & \,({\rm{\Delta }}{H}_{298K}^{0}=-\,27.014\,\mathrm{kcal})\end{array}$$

#### NH_4_Cl/methanol solution

Ammonium chloride, NH_4_Cl, in methanol reacted with CaH_2_ and CaO, then the byproducts leached out as CaCl_2_:

10 $$\begin{array}{rcl}{{\rm{CaH}}}_{2}+2{{\rm{NH}}}_{4}{\rm{Cl}} & \to  & \,2{{\rm{NH}}}_{3}({\rm{g}})+\,2{{\rm{H}}}_{2}({\rm{g}})+{\mathrm{CaCl}}_{2}\ldots \ldots \ldots \ldots ({{\rm{\Delta }}{\rm{G}}}_{298{\rm{K}}}^{0}=-\,56.738\,\mathrm{kcal}),\\  &  & ({\rm{\Delta }}{H}_{298K}^{0}=-\,19.403\,\mathrm{kcal})\end{array}$$

11 $$\begin{array}{rcl}\mathrm{CaO}\,+\,2{{\rm{NH}}}_{4}{\rm{Cl}} & \to  & {\mathrm{NH}}_{3}({\rm{g}})+{\mathrm{NH}}_{4}{\rm{OH}}({\rm{l}})+{{\rm{CaCl}}}_{2}\ldots \ldots \ldots \ldots ({{\rm{\Delta }}{\rm{G}}}_{298{\rm{K}}}^{0}=-\,2.309\,\mathrm{kcal}),\\  &  & ({\rm{\Delta }}{H}_{298K}^{0}=14.698\,\mathrm{kcal})\end{array}$$

The methanol-solubility of NH_4_Cl powder and CaCl_2_ obtained at 25 °C was 3.54 g and 29.2 g per 100 g of methanol, respectively. Additionally, NH_4_OH was well soluble with 31.3 g/100 g methanol^64^. The solubility of hydrogen gas, which led to rare earth magnet deactivation, was much lower in methanol than in water^65^. Compared to the two cases above, the lower enthalpy change showed that Equations (10 and 11) occurred favorably to reduce hydrogenation of Sm_2_Co_17_. Hence, the byproduct was removed perfectly by rinsing with methanol without any damage to Sm-Co. That is to say, the combination of NH_4_Cl/methanol was a unique solution for the chemoselective dissolution of Sm_2_Co_17_ nanostructures.

### Effect of NH4Cl concentration and dissolution times

**Figure 7:**
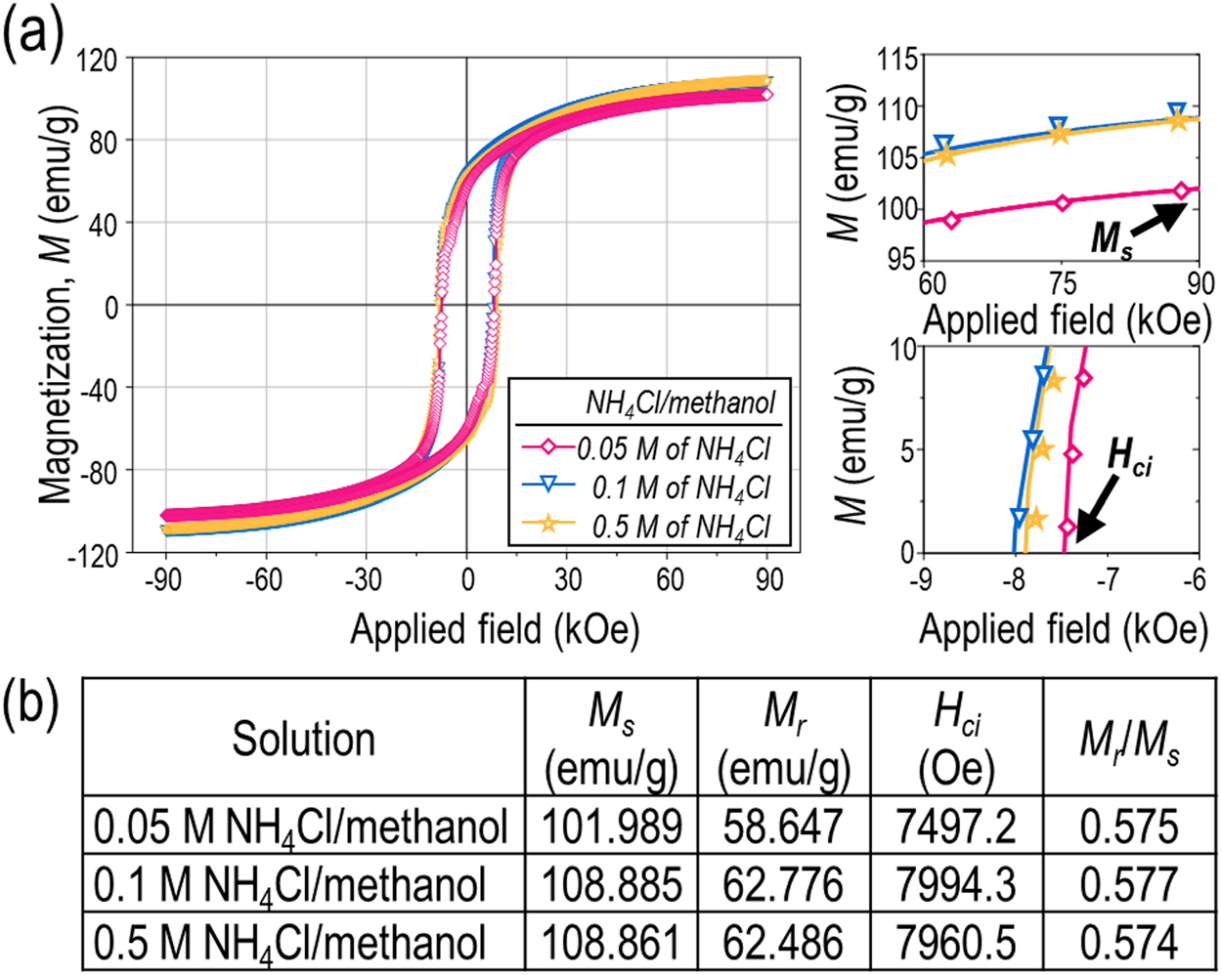
(**a**) Magnetic hysteresis loops of the NH_4_Cl/methanol solution-treated Sm_2_Co_17_ nanofibres with different NH_4_Cl concentrations. (**b**) The corresponding saturation magnetisation (*M*_*s*_), remanence (*M*_*r*_), coercivity (*H*_*ci*_), and squareness (*M*_*r*_/*M*_*s*_) values for the as-washed Sm_2_Co_17_.

With a view to confirm the effect of solution concentration on CaO removal, the NH_4_Cl content was varied from 0.05 M to 0.5 M, corresponding to the maximum concentration in methanol^66^. FE-SEM micrographs and X-ray diffraction patterns of the Sm-Co nanofibres treated with different solution concentrations can be seen in Supplementary Figs. S3 and S4. When a relatively low solution concentration was used (0.05 M of NH_4_Cl) a large quantity of CaO existed without complete removal, indicating an insufficient dissolution source as explained in Equation (11). Magnetic hysteresis curves of the solution-treated Sm-Co nanofibres can be seen in Fig. 7(a). As the concentration increased from 0.05 M to 0.1 M, *M*_*s*_ likewise increased from 102.0 emu/g to 108.9 emu/g and *H*_*ci*_ increased from 7497.2 Oe to 7994.3 Oe. When the concentration was further increased to 0.5 M, all magnetic properties were retained. Likewise, the effect of a 10 to 120 min dissolution time on the X-ray patterns of treated samples showed no distinct difference (see XRD patterns in Fig. S5). Therefore, higher NH_4_Cl concentrations and increased treatment durations did not enhance the magnetic properties of the hard-phase material while selectively rinsing away byproducts.

### Applicability of the chemoselective dissolution effect on other hard magnetic phases

As mentioned previously, a near theoretical value of *M*_*s*_ was a clear manifestation of a highly pure single-phase magnet as verified from XRD, TEM, and ICP-AES analysis. To discuss the applicability of the chemoselective dissolution effect on not only Sm_2_Co_17_ but also other rare earth based magnetic phases (*e.g*., SmCo_5_, Nd_2_Fe_14_B) from a comparison of *M*_*s*_, we summarized the experimental *M*_*s*_ values obtained from the current study, and other literature for each hard magnetic phase in Table 1. *H*_*ci*_ values were not included because coercivity is ruled by extrinsic conditions such as the shape, size, and microstructure of the magnet^67^. All the listed nanomaterials were synthesized *via* calcium thermal reduction and were composed of a single-phase and obtained after solution-dissolution in their own particular way. It could be seen that even considering a difference in treatment and measurement conditions, the highest *M*_*s*_/*M*_*bulk*_ ratio was only achieved with NH_4_Cl-treated samples, which was over 80%, while most of the other cases achieved small *M*_*s*_ values of less than half *M*_*bulk*_. Furthermore, when the same solution-treatment processes were considered for samples with the same phase, a similar level of *M*_*s*_ was obtained regardless of synthetic method. It was an interesting and convincing demonstration that no matter how the nanostructured magnets possessed a “dead” layer on their surface, a substantial *M*_*s*_ loss was obvious due to side products derived from chemical interactions during the rinse processes for Ca removal. With this regard, chemoselective dissolution with NH_4_Cl played a decisive role toward the preparation of high-purity nanomagnets. However, further investigations and experiments are needed for other intermetallic systems; the possibility of *M*_*s*_ improvements for Sm-Co and Nd-Fe-B systems have been tentatively confirmed as summarized from this comparison.

**Table 1:** Summary of the saturation magnetisation (*M*_*s*_) values of chemically synthesized rare earth-based magnetic nanomaterials with various treatment solutions in our study and other literature.

Materials and their *M*_*bulk*_ values^15^^b^	Treatment solution for CaO and leftover reductant removal	Magnetic properties		Reference
		*M*_*s*_ (emu/g)	*M*_*s*_ */M*_*bulk*_	
SmCo_5_ (107.3 emu/g)^a^	Distilled water	42.5	39.60%	^20^
		~43	40.00%	^11^
		59.424	55.40%	This work^c^
	Distilled water & dilute hydrochloric acid solution	43.65	40.70%	^68^
		~55	51.30%	^29^
	Distilled water & anhydrous ethanol	60	55.90%	^69^
	Dilute acetic acid solution	88.315	82.30%	This work^c^
	NH_4_Cl/methanol solution	100.78	93.90%	
Nd_2_Fe_14_B (168.0 emu/g)^a^	Distilled water	40.586	24.20%	This work^d^
		55	32.70%	^22^
	Distilled water & dilute acetic acid solution	71.4	42.50%	^24^
		75	44.60%	^23^
	NH_4_Cl solution	135.67^b^	80.80%	^44^
		138.88	82.70%	This work^d^
Sm_2_Co_17_ (114.0 emu/g)^a^	Distilled water & dilute acetic acid solution & anhydrous ethanol	~65	57.00%	^25^
	Dilute acetic acid solution	74.4	65.30%	This work
	Distilled water	85	74.60%	^70^
		~91	79.80%	This work
	NH_4_Cl/methanol solution	~108.9	95.50%	This work

^a^Theoretical value for the bulk material.

^b^For the sake of convenient comparison, values previously reported in SI units (*i.e*., A/m, Tesla (T)) were converted to Gaussian units of emu/g.

^c^See Supplementary Fig. S7.

^d^See Supplementary Fig. S8.

## Conclusion

In this study, we examined the magnetic properties of calcium-reduced Sm_2_Co_17_ nanofibres prepared with various treatment solutions (*i.e*., distilled water, dilute acetic acid, sugar solution, and NH_4_Cl/methanol solution) and discussed the effects of chemoselective dissolution on the purity, surface microstructure, and magnetic characteristics of Sm-Co. A study was performed comparing the calculated thermodynamic parameters such as the change in Gibbs free energy (∆G°) and enthalpy (∆H°) for each reaction at room temperature. Despite utilizing the same Sm_2_Co_17_ nanofibres as starting powder materials, obvious enhancements to *M*_*s*_ (about 108.885 emu/g) near the theoretical value and high *H*_*ci*_ (about 7994.3 Oe) were clearly obtained *via* chemoselective reactivity of NH_4_Cl/methanol solutions without yielding any damage to Sm-Co; however, other water-based solutions led to the formation of side products or rare earth magnet deactivation. There was no observed time-dependency or concentration-dependency with NH_4_Cl. Compared to previously reported studies, we deduced that the combination of a calcium-assisted thermal reduction and subsequent treatment with NH_4_Cl/methanol was the key toward achieving high purity and thus high magnetic properties. This concept is expected to overcome the inevitable property loss of all calcium-reduced materials and can be extended to other magnetic materials obtained through calcium thermal reduction processes and may help to prepare high-purity phases as raw materials for exchange-coupled magnets.

## Methods

### Chemicals

Calcium hydride granules [CaH_2_, 92%, Alfa Aesar, England], acetic acid [CH_3_COOH, 99.9%, DAEJUNG Chemical & Metals Co., Ltd., South Korea], sucrose powder [C_12_H_22_O_11_, 99.5% up, Sigma-Aldrich, St. Louis, MO, USA], ammonium chloride [NH_4_Cl, 99.99%, Sigma-Aldrich], anhydrous methanol [CH_3_OH, 99.9% up, Sigma-Aldrich], and acetone [CH_3_COCH_3_, 99.9% up, Sigma-Aldrich] were utilized as raw materials. Distilled water was produced and used from a reverse-osmosis system [RO2000, Nexpure®]. All chemicals were used as is without further purification.

### Preparation of calcium-reduced hard magnetic nanostructures

As a hard magnetic material, Sm_2_Co_17_ was comparatively easier to prepare than three-element systems such as Nd-Fe-B. Sm_2_Co_17_ nanofibre synthesis was performed through a procedure modified from our previous work that involved electrospinning and several annealing processes^12^. The precursor fibres consisting of Sm_2_O_3_ and fcc-Co were mixed with CaH_2_ granules (CaH_2_/as-prepared nanofibre = 2 (*vol*.)) and the reduction-diffusion (R-D) process with CaH_2_ was performed at 700 °C for 3 h in argon gas (see the morphology, size distribution, and phase transformation of the as-synthesized Sm_2_Co_17_ nanofibres in Fig. S1). After the reduction, most residual CaH_2_ granules were sifted through a fine 16 mesh sieve in a glove box under nitrogen. The isolated powder was stored in a vacuum desiccator.

### Preparation of solution mixtures for chemoselective dissolution

Four different solutions were chosen: pure distilled water, dilute acetic acid, sucrose juice, and a NH_4_Cl/methanol solution (see the results and discussion, for background with regard to the selection). The aqueous acidic solution and NH_4_Cl solution were prepared by dissolving an appropriate amount of each reagent to reach a concentration of 0.1 M, respectively. 85 *w*/*v*% of sucrose solution, where *w/v* (%) = [mass of solute (g)/volume of solution (mL)] × 100, was prepared by mixing 850 g of sucrose powder with an adequate amount of water in 1000 mL of solution.

### Chemoselective dissolution procedure

0.1 g of calcium-reduced Sm_2_Co_17_ nanopowder was added and mixed into 100 mL of each solution for 30 min at 25 °C using a shaking incubator (60 rpm); the mixtures were centrifuged at 8000 rpm for 20 min to separate the solid powder from the solutions. The obtained Sm-Co samples were mixed with 100 mL of each fresh solution and filtered again. All solutions were collected for elemental analysis. The nanofibres were finally rinsed with acetone to remove any residual solution and were stored in a vacuum oven until characterized to limit partial oxidation and any unexpected reactions.

### Characterization

*HSC Chemistry* software was employed to calculate thermodynamics parameters such as the change in Gibbs free energy and the enthalpy for reactions between chemicals and solutions. Field-emission scanning electron microscopy [FE-SEM, MIRA-3, Tescan] and transmission electron microscopy [TEM, JEM-2100F, JEOL] were employed to analyse the morphology, surface characteristics, and microstructure of the surface treated Sm-Co nanofibres. The phase and crystallographic characteristics of the fibres were identified using an X-ray diffractometer [XRD, D/MAX-2500/PC, Rigaku] with Cu Kα radiation (1.5406 Å). The concentrations of three major elements (*i.e*., Ca, Sm, and Co) within the nanofibres and solutions obtained after the chemoselective process were determined *via* transmission electron microscopy, energy-dispersive X-ray spectroscopy [TEM-EDS, JEM-2100F, JEOL], and inductively coupled plasma optical emission spectroscopy [ICP-OES, Optima 8000, PerkinElmer], respectively. Magnetic properties were measured at room temperature using a physical property measurement system [PPMS, PPMS-9T, Quantum Design] with up to 9 tesla.

## Electronic supplementary material

Supplementary information

## Data Availability

All data generated or analysed during this study are included in this published article and its Supplementary Information files.
