# Supplementary material for: Near theoretical ultra-high magnetic performance of rare-earth nanomagnets via the synergetic combination of calcium-reduction and chemoselective dissolution
Source: Sci Rep. 2018 Oct 23;8:15656. doi: 10.1038/s41598-018-33973-z (PMC6199341; doi:10.1038/s41598-018-33973-z)
Supplement: Supplementary file 1 — Supplementary information [file 41598_2018_33973_MOESM1_ESM.pdf]

Supplementary Information for:

Near theoretical ultra-high magnetic performance of rare-earth nanomagnets *via* the synergetic combination of calcium-reduction and chemoselective dissolution

Jimin Lee[1], Tae-Yeon Hwang[2], Hong-Baek Cho[1],
Jongryoul Kim[1], and Yong-Ho Choa[1,2,*]

[1]Department of Materials Science and Chemical Engineering, Hanyang University, 55, Hanyangdaehak-ro, Sangnok-gu, Ansan-si, Gyeonggi-do 15588, Korea
[2]Department of Fusion Chemical Engineering, Hanyang University, 55, Hanyangdaehak-ro, Sangnok-gu, Ansan-si, Gyeonggi-do 15588, Korea

*Corresponding author:
choa15@hanyang.ac.kr, Tel: +82-31-400-5650, Fax: +82-(31)-418-6490 (Yong-Ho Choa).

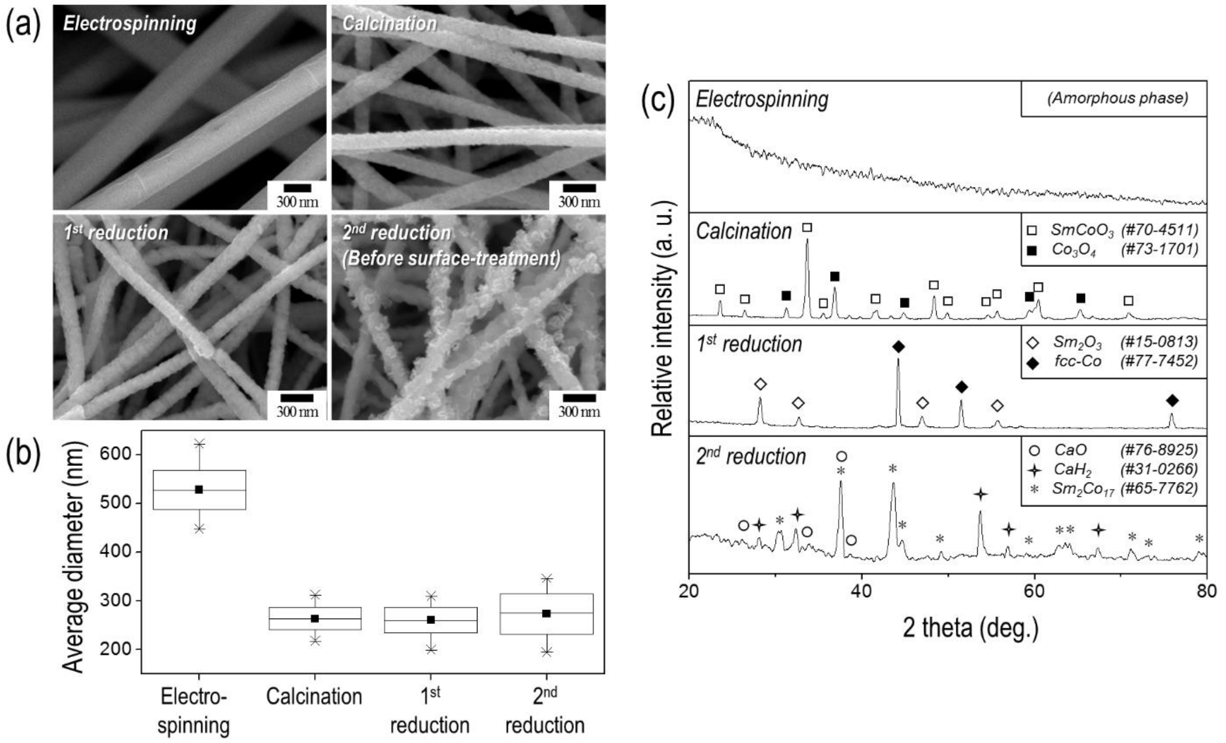

Figure S1. (a) Surface morphologies, (b) variations in fibre diameter, and (c) crystallinity of the series of Sm-Co based fibres obtained during the synthesis of the Sm_2Co_{17} nanofibres.

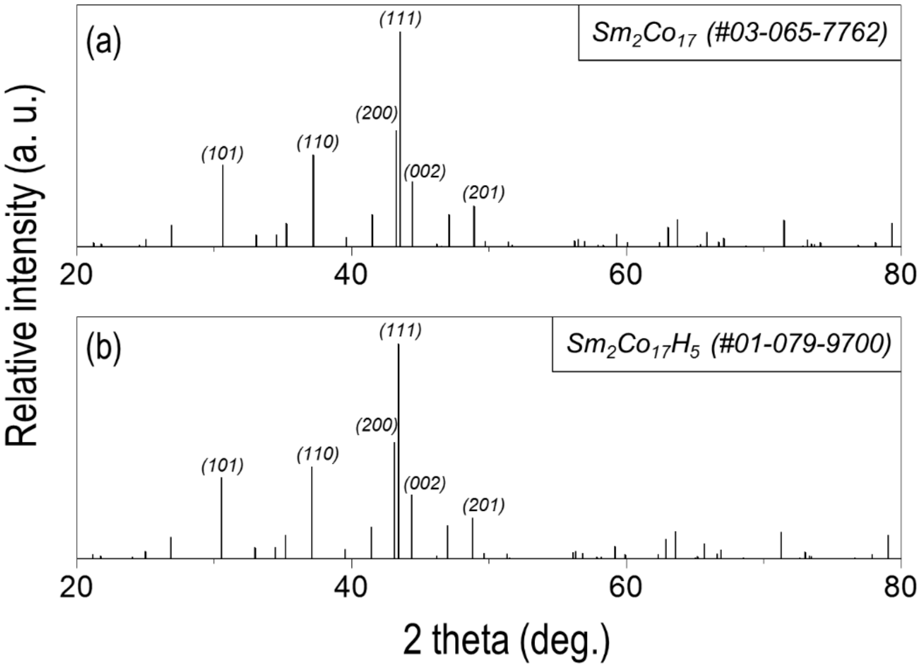

Figure S2. Reference XRD patterns of (a) Sm_2Co_{17} (JCPDS#03-065-7762) and (b) $Sm_2Co_{17}H_5$ (JCPDS#01-079-9700), respectively.

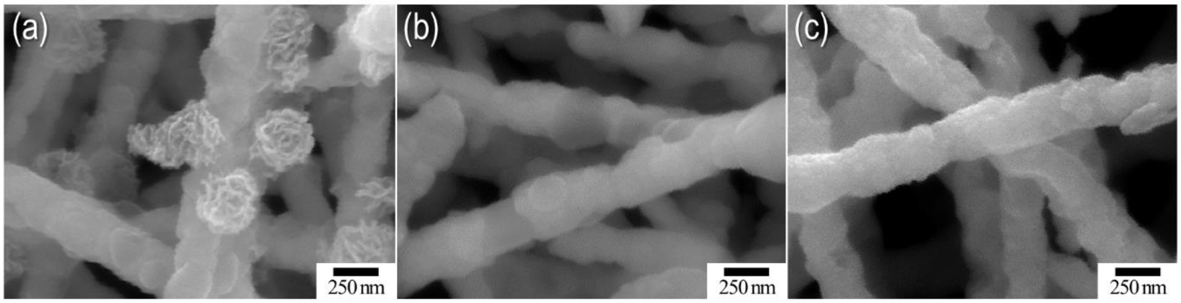

Figure S3. FE-SEM micrographs of the NH_4Cl/methanol solution-treated Sm_2Co_{17} nanofibres with different NH_4Cl concentrations: (a) 0.05 M, (b) 0.1 M, and (c) 0.5 M.

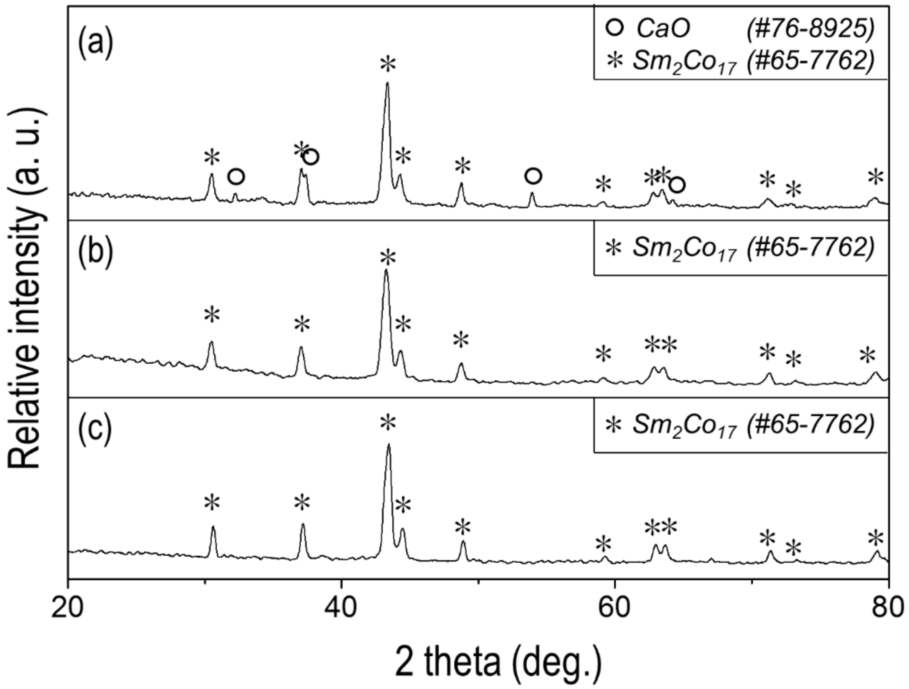

Figure S4. XRD patterns of the NH_4Cl/methanol solution-treated Sm_2Co_{17} nanofibres with different NH_4Cl concentrations: (a) 0.05 M, (b) 0.1 M, and (c) 0.5 M.

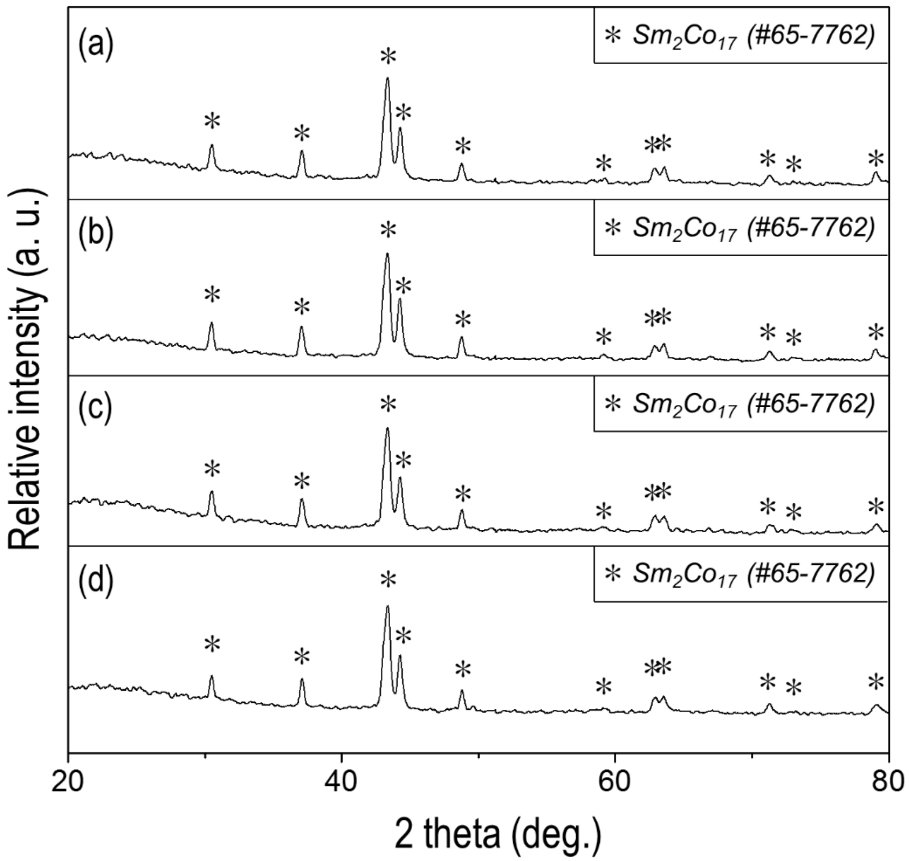

Figure S5. XRD patterns of the 0.1 M NH_4Cl/methanol solution-treated Sm_2Co_{17} nanofibres with different dissolution periods: (a) 15 min, (b) 30 min, (c) 60 min, and (d) 120 min.

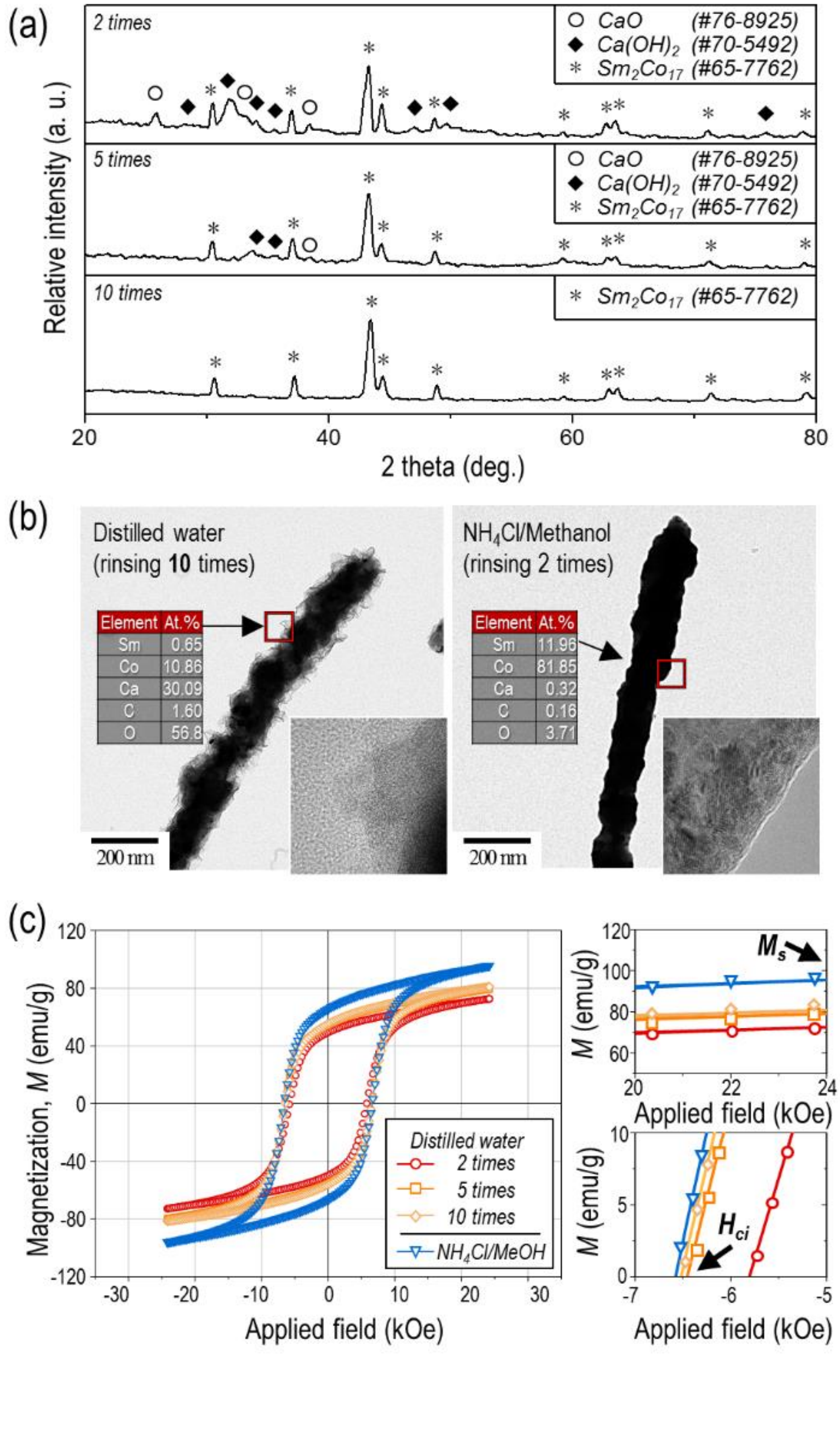

Figure S6. (a) XRD patterns, (b) TEM micrographs, and (c) magnetic hysteresis loops of the distilled water-treated Sm_2Co_{17} nanofibres with different dissolution times.

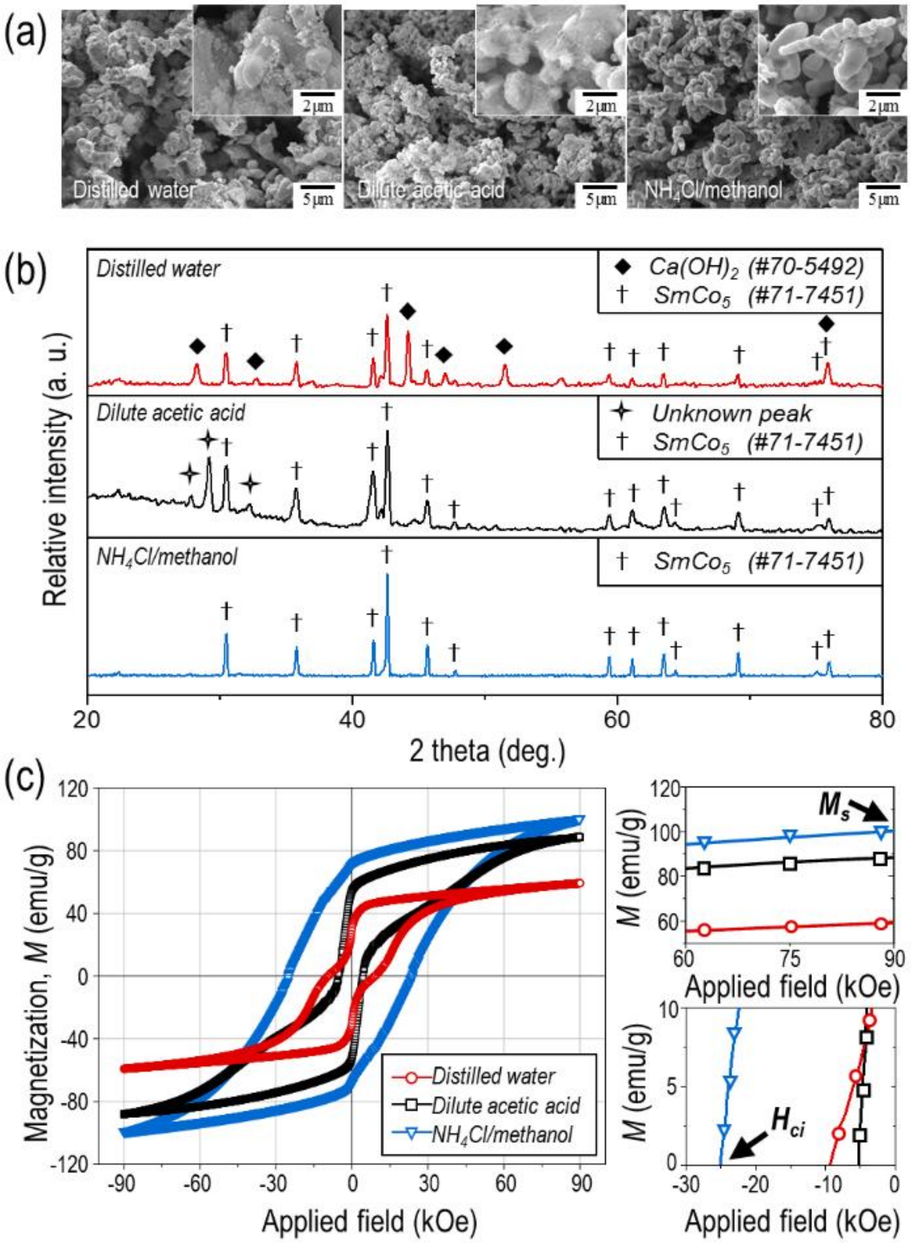

(d)

Solution	M_s (emu/g)	M_r (emu/g)	H_{ci} (Oe)	M_r/M_s
Distilled water	59.424	30.195	9412.0	0.508
Dilute acetic acid solution	88.315	45.318	5261.9	0.513
NH_4Cl/methanol	100.780	72.526	25120.0	0.720

Figure S7 (a) FE-SEM micrographs, (b) XRD patterns, (c) magnetic hysteresis loops and (d) the corresponding magnetic parameters of the as-washed $SmCo_5$ nanoparticles.

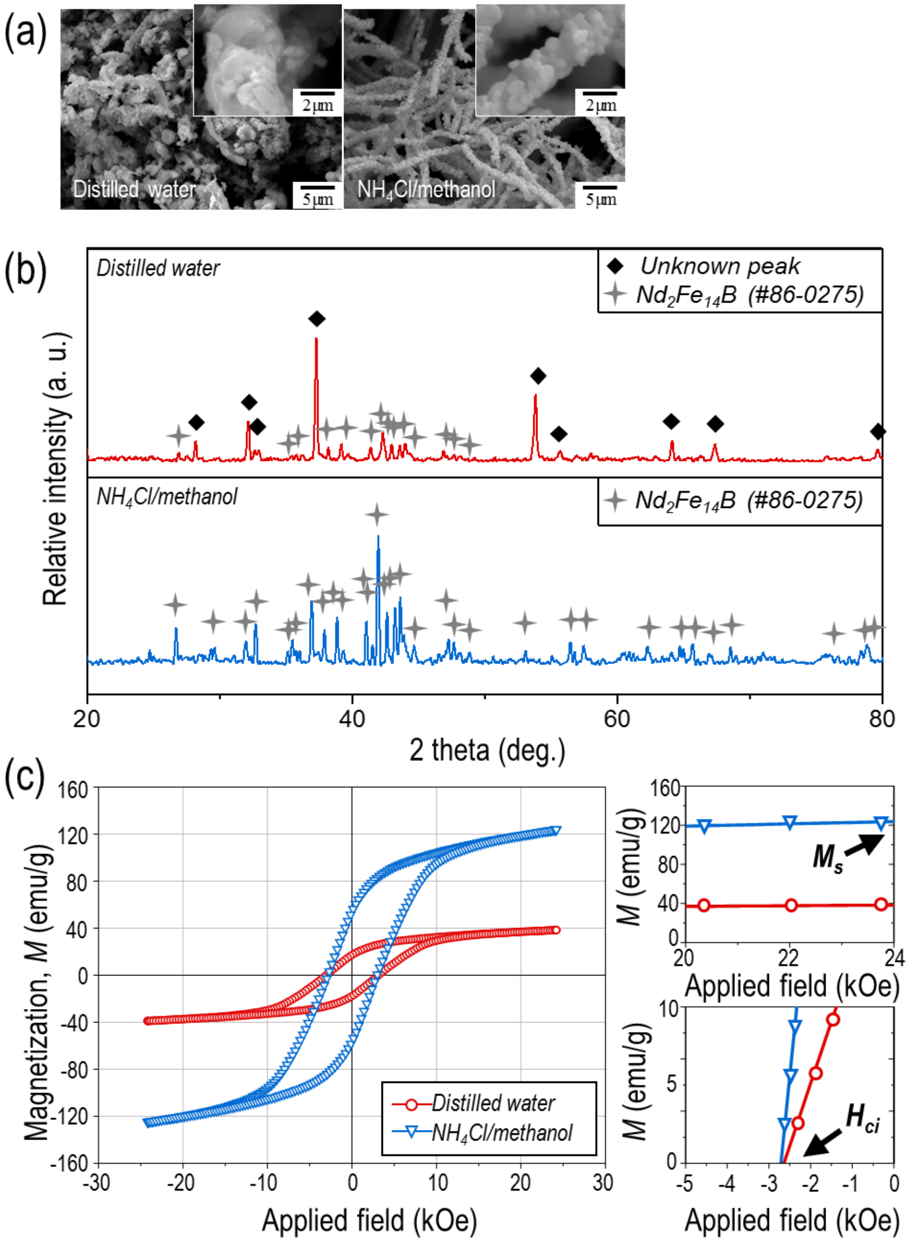

(d)

Solution	M_s (emu/g)	M_r (emu/g)	H_{ci} (Oe)	M_r/M_s
Distilled water	39.495	17.003	2688.1	0.431
NH_4Cl/methanol	124.127	54.418	2780.3	0.438

Figure S8 (a) FE-SEM micrographs, (b) XRD patterns, (c) magnetic hysteresis loops and (d) the corresponding magnetic parameters of the as-washed $Nd_2Fe_{14}B$ nanofibres.
